# *“But you have to start somewhere….”*: Nurses’ perceptions of what is required to provide quality neonatal care in selected hospitals, Kenya

**DOI:** 10.12688/wellcomeopenres.15592.2

**Published:** 2020-02-17

**Authors:** Mary Nyikuri, Pratap Kumar, Caroline Jones, Michael English

**Affiliations:** 1 Strathmore University Business School, Strathmore University, P.O. Box 59857 – 00200, Nairobi, Kenya; 2 KEMRI-Wellcome Trust Research Program, P.O. Box 43640 – 00100, Nairobi, Kenya; 3 Nuffield Department of Clinical Medicine, Centre for Tropical Medicine, University of Oxford, Oxford, UK

**Keywords:** Neonatal nursing, work environment, Quality of care, Ethnography, Kenya

## Abstract

**Background:** Kenya has one of the highest rates of neonatal mortality in the world at 22/1,000 live births. Improving the quality of newborn care would greatly improve survival rates. There is an increasing consensus that strong health systems are key to achieving improved health outcomes. However, there is significantly less agreement on what to strengthen in low and middle-income countries such as Kenya. As nurses are the main caregivers in many inpatient settings, efforts aimed at improving the quality of facility care for sick newborn babies need to take into account nurses views and opinions. Our intent in this paper is to describe the current state of the nursing environment and what would be required to improve the quality of those environs from nurses’ perspectives.

**Methods**: Between January 2017 and March 2018, we collected data through non-participant observations, unsolicited conversations and review of admission registers. We also conducted 29 individual in-depth interviews with nurses working in the newborn units (NBU) of a public sector hospital (n=10), a private sector hospital (n=11) and a faith-based hospital (n=8).  The interviews were digitally audio recorded, transcribed verbatim and, together with observation notes, analysed using thematic content analysis.

**Results: **Nurses as frontline care givers and intervention intermediaries, irrespective of their work contexts, have similar aspirations, needs and expectations from the health systems of how they should be supported to provide quality inpatient care for newborns. These are about the structure of the work environment, especially human resources for health, and the consequences of inadequate structure. They are also about how care is organised and systems that respond to emergencies.

**Conclusion: **Interventions and investments to improve quality need to be directed towards experienced based co-design where we listen to the problems that nurses experience.

## Introduction

Kenya has in the region of 29,000 neonatal deaths per year and, although some progress has been made in reducing neonatal mortality, the rate remains high at 22/1000 live births ^1^. Research indicates that improving newborn survival will require better quality hospital services ^2– 6^. Further research has shown that constraints in low- and middle-income countries, such as shortages in the health workforce and a lack of the required infrastructure, undermine delivery of effective interventions to tackle neonatal mortality ^6^. These reports exploring the existing quality of newborn care have typically focused on the ‘hardware’ of health systems, assessed using inventories, or aspects of the process of medical care assessed as adherence to clinical guidelines ^7– 9^. Globally, including in Kenya, the majority of inpatient facility based care is provided by nurses ^10, 11^, yet their voices on their experience of quality are seldom heard. In particular, their opinion on what the structural requirements are to deliver quality care is absent. The result is a gap in our understanding of key concerns that affect nurses and how local context shapes care. This study adopted a qualitative approach to go beyond quality as an inventory of hardware to include organisational context by delving into nurses ‘lived’ experience of their context of care. The aims of this study were to explore and describe the structural context surrounding nursing care in selected Kenyan newborn units and to document nurses’ perceptions of what matters to them to provide quality care for hospitalised sick newborns. In Box 1, the authors describe what this study adds to the field of neonatal nursing.

**Box 1. Value of the study**

**What is already known?**

   •   Kenyan hospitals vary in the structural resources available to them to provide quality inpatient care to sick newborns when assessed using typical resource checklists, and resource availability may be linked to the ownership of facilities (public, private for profit and private not-for-profit).

**What are the new findings?**

   •   Although nurses work in different service infrastructures and organisational contexts, they have similar views on what hospital capacities are required for them to provide quality care to inpatient newborns.

   •   Resource scarcity identified through surveys such as Service Provision Assessments and Service Availability and Readiness Assessments tell a very partial story on how structure may affect nurses’ work and the babies they care for; a more complete understanding is gained be exploring what they themselves can tell us.

**What do the new findings imply?**

   •   Interventions and investments to improve quality need to be directed towards experienced based co-design, where we listen to the things that nurses tell us are problems. Problems such as infrastructure, staffing and interpersonal team work if we are to improve hospital services for newborns

## Materials and methods

### Ethical statement

The research received ethics clearance from KEMRI Scientific Ethics Review Committee (certificate no. KEMRI/SERU/CGMR-C/SU-IRB 0060/16/3555). Permission was also sought from participating hospitals as well as the Nairobi County Department of Health. All participants provided voluntary written consent before being interviewed

### Study setting

In Kenya, health services are provided by the government (henceforth referred to as public), private for profit (private) and private ‘not for profit’ institutions (referred to as faith-based) ^12^. The public sector provides approximately 50–60% of the health services ^13, 14^. This study was undertaken in the newborn units (NBUs) of one public hospital, one faith-based hospital and one private hospital, all located in Nairobi County, Kenya. The public hospital was selected as it is the only formerly district level facility that provides first level referral services to primary care facilities and this study was looking to engage a government-run district equivalent facility ^5^. The other two facilities were purposively selected to represent the faith-based and private sectors based on their annual admissions of >500 newborns and their willingness to participate in the study. The public hospital spreads across buildings constructed in the colonial era and at the time of this study, the maternity and newborn wards were located in the only two-storey building. Between 1st July 2014 to 30th June 2015, the hospital recorded 1006 newborn inpatient admissions, accounting for 6.8% of all newborn admissions in Nairobi County and 10.6% of all public sector facilities ^7^. The faith-based hospital began its operations in the year 2000, and between 1st July 2014 to 30th June 2015, it recorded 1435 newborn inpatient admissions, accounting for 9.7% of all newborn admissions in Nairobi County. The private hospital formally began post-graduate teaching in July 2005 and its inpatient services extend to provision of tertiary care. Between 1st July 2014 to 30th June 2015, it registered 657 newborn inpatient admissions, accounting for 4.5% of all newborn admissions in Nairobi County. With devolution of health services, where the county governments are responsible for service delivery, the hospitals are as diverse as the leadership and governance systems in the counties. However, most public hospitals are characterised by overcrowding and high patient to nurse ratios. The Private hospital represents majority of high end private hospitals where facilities are well maintained, no overcrowding and sufficient staffing levels. The faith based hospital on the other hand is not representative of faith based hospitals as this sector is as diverse as the founder and funders.

According to the American Academy of paediatrics’ classification if levels of neonatal care, the Public and faith based hospital NBU offers level II neonatal care while the private hospital offers level IV care ^15^.

### Data collection

This study adopted an ethnographic qualitative research design. Data were collected between January 2017 and March 2018. The first author (MN), who is female and was a PhD student at the time of the study, carried out non-participant observations and took field notes. The total time of observations were 78 hours in the public hospital and 46 hours and 88 hours in the private hospital and faith-based hospitals, respectively. In each location, the first author began by establishing rapport with nurses and other health care workers on the ward. The aim of this rapport building was to help nurses relax as well as understand the aims and objectives of the research and also for the researcher to better understand the work spaces and later to triangulate what nurses did in relation to what they said in interviews. The researcher also engaged in informal conversations to help her clarify what was happening, mainly during day shifts when many processes of care were carried out. In addition, 29 face to face in-depth semi-structured interviews were conducted with 10 nurses from public, 11 from private and 8 from faith-based hospitals through homogeneous purposive sampling where all nurses working in the NBU were eligible for inclusion. To allow for an equal number of nurses across sites, we endeavoured to interview at least half of the total number of nurses in the public and private hospitals. The first author interviewed all nurses in the faith-based hospital, 10/18 in the public hospital and 11/20 in the private hospital. The interviews, which lasted between 45–60 minutes, were conducted at a time and place chosen by the nurses, which was mostly a quiet room at the hospital. The interview guide, which had the guiding question ‘Can you tell me what quality care is?’ with probes built in for clarification, was pilot tested for clarity and logical flow among nurses working in the same institution as the first author (interview guide is provided as *Extended data*) ^16^. Audio recordings were taken of 26 of the interviews, and subsequently transcribed verbatim by the first author MN. Six interviews which were conducted in a mixture of English and Kiswahilithe national language, were translated into English for ease of understanding among co-authors. To maintain quality of data during translation, MN did careful comparison with the original interview questions and against other transcripts. In addition, MN carried out back translation to ensure that original meaning was maintained. Detailed notes were taken during the interviews with the three nurses who declined to be recorded and later expanded.

### Data analysis

Analysis began as soon as the first few interviews were done by regular discussions among co-authors and once saturation had been established, the first author, MN, carried out analysis by open coding using Nvivo 10 software ^17^. In the absence of Nvivo which is not open source, an open source software such as FreeQDA could also be used. Initial codes were generated from interview guide whilst additional emerging themes were subsequently analysed in relation to relevant literature, culminating in thematic analysis ^18^. The first author generated matrices of all relevant codes among nurses and across health sectors for easy comparison. These were reviewed by all co-authors, resulting in merging of similar codes into main themes that were then used for presentation of the results.

The findings have been reported using the COREQ checklist (see *Extended data*) ^16^.

### Rigour

To ensure data quality, and analytic rigour, the interviews were first triangulated with observation data; secondly by general feedback of the themes to the nurses for sense checking; thirdly, the researcher made presentations in various local forums where feedback was received and incorporated.

## Results

### Demographic characteristics of the interviewees

The majority of the nurses were aged between 30 and 39 years. Nearly half of the nurses had spent more than 10 years in their current facility; only one had more than 10 years’ experience in the NBU and most had between one and six years’ experience in this role ( Table 1).

**Table 1.** Demographic characteristics of the participants.

Characteristic	Sector		
	Public	Private	Faith-based
Age distribution			
20–29	0	1	4
30–39	4	8	2
40–49	5	2	2
50 and above	1	0	0
Training level			
Diploma	8	4	6
Higher Diploma	2	0	1
Bachelors	0	6	1
Masters	0	1	0
Years in current facility			
Less than 1 year	0	0	1
1–3	0	3	3
4–6	1	3	2
7–9	2	1	1
10 and above	7	4	1
Years as NBU nurse			
Less than 1 year	2	0	1
1–3	3	4	5
4–6	4	4	2
7–9	1	2	0
10 and above	0	1	0

NBU, newborn unit.

After coding all the interviews and observation data, the researchers found that the key issues were the relationship between ‘structural’ elements of nurses’ context and their work; that is, how structural issues shape quality in practice. The nurses have a fairly consistent view of what quality care is in theory.

In their definition of quality, they described why infrastructure matters and how it goes beyond having or not having something. We first present in section I the observed work environment; the real picture of the spaces where nurses work and the variation across these contexts, a variation that can easily be missed through inventories, which may classify very different places as similar on the basis of the list of ‘items’ they contain. In section II, we present what nurses perceived as the necessary capacities (what needs to be changed) for them to provide quality neonatal care.

## Section I: A description of the NBUs

### The layout and organisation of the NBU in the public hospital

**Figure 1.**
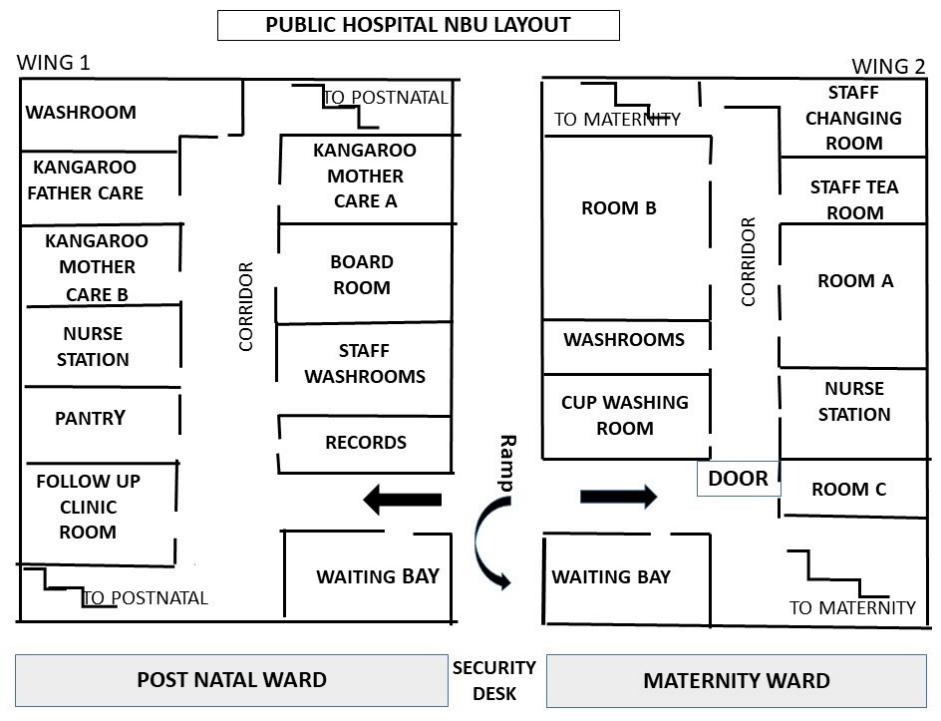
The newborn unit in the public hospital.

The public hospital’s NBU is housed on top of the maternity ward. It is rectangular in shape with a corridor dividing the space into two wings A and B, which are further divided by concrete walls into smaller spaces that are designated for various services ( Figure 1). Three of these rooms (A, B & C) located in wing B are used for accommodating babies at different care levels based on the intensity of nursing required, as described in Table 2. Rooms A, B and C are all extremely overcrowded: room A has a total floor space of 360 ft ^2^ and contains nine infant incubators; room B has a total floor space of 450 ft ^2^ and houses 18 baby beds; and room C has a total of 150 ft ^2^ and holds four incubators.

**Table 2.** A description of the newborn unit layout in the public hospital.

Room Label	Room description	Contents
Room A	Houses preterm and very sick babies	Contains a resuscitation section; nine incubators arranged along the walls with no space between; and a wooden table and benches on both sides where different cadres of health care personnel sit to make reports.
Room B	Houses stable babies	It has 18 baby cots, although four are broken and therefore sometimes used for storing dirty linen; has a wooden table and two benches that are used by different cadres of health care personnel for paperwork; there are three waste disposal bins close to the entrance.
Room C	Referred to as the isolation room, housing babies admitted from outside the hospital	Has four incubators with no space between
Nurse station in wing B	Acts as the nurse office	The walls have laminated copies of standards and guidelines of various newborn care procedures; shift rota and internal memos; a desk and two chairs; a lockable drawer with stationery and patient files.
Tea room	Nurses and other health care personnel have tea and make phone calls	Has a water dispenser; a lockable metallic locker where the nurse in- charge keeps all medical supplies as well as cot linen; a table and three chairs; two thermos flasks and several cups.
Kangaroo mother (KMC) and father (KFC) care rooms	These rooms were established with the support of an international non- governmental organisation	Each has a sink, a table in the corner with hot water and an assortment of beverages for the mothers and fathers. KMC rooms A & B have seven beds each, while the KFC room has three beds.
Board room	For meetings and continuous medical education	Two large tables, office and plastic chairs
Follow-up room	For babies that have returned two weeks post discharge for follow-up	Has a desk, weighing machine, two chairs
Pantry room	Storage for linen	Clean linen and cleaning materials

The NBU has three kangaroo care rooms located in wing A, which were equipped with the support of an international non-governmental organization.

In addition to the congestion witnessed as a result of fitting many incubators into the small spaces, the presence of babies’ mothers or guardians, as well as nursing, clinical and nutrition students compounded the overcrowding. There are instances when more than four training institutions can have their students in this ward involved in different medical procedures or sitting at the table reviewing patient files.

Although the NBU is located in a building constructed three years before this study, there are often problems related to poor maintenance. It was common to see leaking taps tied with nylon strips, exposed electricity wires, dysfunctional sockets, broken furniture, and dirty linen stacked in broken cots. The nurse in charge reported that at least once a month, the ward experiences extremes of water shortages as well as flooding. Whenever there is no water, there is a foul smell from the toilets. Flooding often happens at night because there is leakage due to rusty taps.

### The layout and organisation of the NBU in the private hospital

The private hospital has two levels of care for sick newborns. The first level is the intensive care unit (ICU), housing babies requiring close monitoring and high level interventions including surgery. The second level is the high dependency unit (HDU), housing newborns requiring specialised medical attention and observation around the clock.

**Figure 2.**
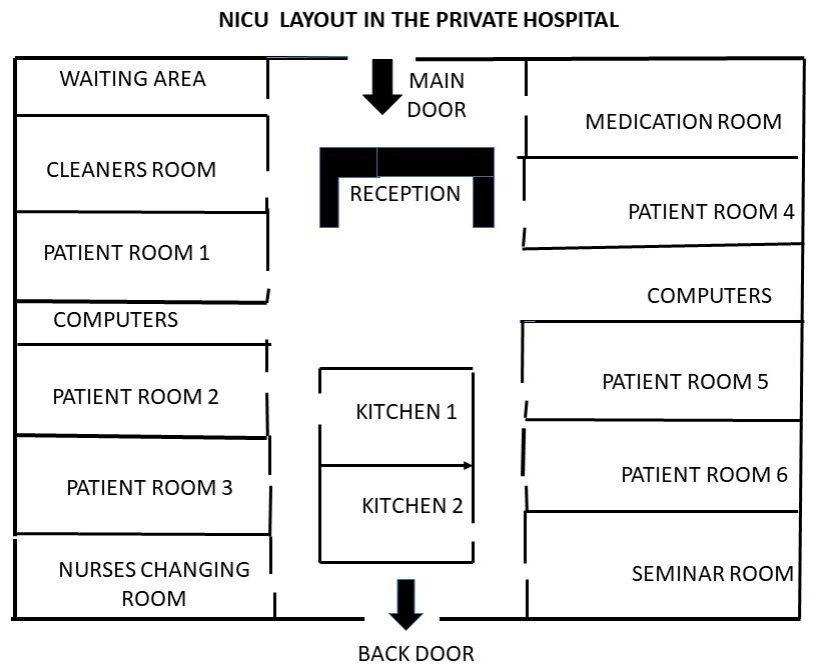
The newborn intensive care unit in the private hospital.

***Newborn Intensive Care Unit (NICU).*** The NICU is part of a broader adult and paediatric ICU, which together is comprised of six patient, administrative and other facility rooms ( Figure 2). The NICU has three dedicated rooms, labelled 4, 5 and 6 in Figure 2. However, during times of increased newborn admissions, there is the possibility of expanding into the other ICU beds. It is a highly regulated environment for both visitors and health care personnel. Access was controlled through both electronic and manual mechanisms.

Each patient room has a floor space of 432ft ^2^, accommodating two babies, and can therefore accommodate two families and one nurse. Figure 2 depicts the floor layout of the intensive care unit.

The reception that serves the whole ICU has a high desk where the nurse in charge and the security guard sit. As visitors approach the reception, there are instructions for them to observe good hand hygiene. It is also furnished with CCTV and monitor screens that display the layout of the entire floor. Some screens indicate movement, while some sound alarms whenever the patient’s vital signs are out of normal measures.

NICU rooms 4, 5 and 6 have two incubators, two monitors, a hand sanitizer above each incubator, a desk, a chair, patient files and a medication trolley. As is observed in the whole ICU, outside each of the patient rooms is a desktop computer that is shared between two nurses. This is mainly used for checking lab results. It is also used either before or at the end of a shift for access to standards, policies and guidelines related to the care of newborns.

The changing rooms and bathrooms for staff are located at the back of the ward, as shown in Figure 2, while on the opposite side of the changing room is the seminar room. The seminar room is used for counselling patients and family members, updating them on the condition of their babies, continuous medical education or general ward meetings. The space gives one a sense of a spacious, well organised room with a high sense of privacy.

***Newborn High Dependency Unit (NHDU).*** The NHDU, which specifically houses newborns, is a severely overcrowded multiple bed ward occupying a total of 22oft ^2^ with seven incubators. There is a nurses’ station, an isolation room that houses babies with a contagious condition and a patient area. A comfortable armchair is placed besides each incubator for mothers. Entry into the ward is also regulated by a security guard, as in the NICU. The nurse station also has computers for use as in the NICU and a mobile phone for communication. Nurses in this ward have no access to their own changing room and have to use one located in the maternity unit, approximately 20 metres away.

**Figure 3.**
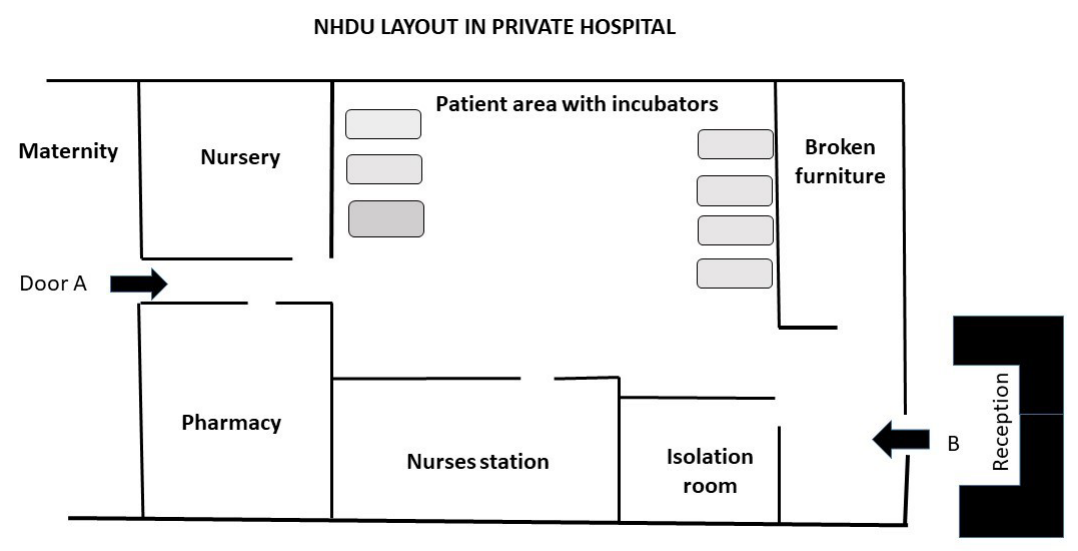
The high dependency unit in the private hospital.

The ward appears and feels organised but small and feels congested around lunch hour when most parents come in to feed their babies. No other visitors except grandmothers and designated next of kin are allowed into this ward. Whenever there is an overflow of patients, they are admitted to the NICU downstairs or some more incubators are fitted ( Figure 3).

### The layout and organisation of the NBU in the faith-based hospital

The NBU is located within the postnatal ward, located at the rear of the hospital on the first floor, two buildings away from the labour ward and three buildings from theatre. Entry into the post-natal ward is through double wooden doors and is controlled by a nurse inside the ward at the nurse station. Visitors into this general post-natal ward are not required to take off their shoes, outer clothing or wash or sanitize their hands.

**Figure 4.**
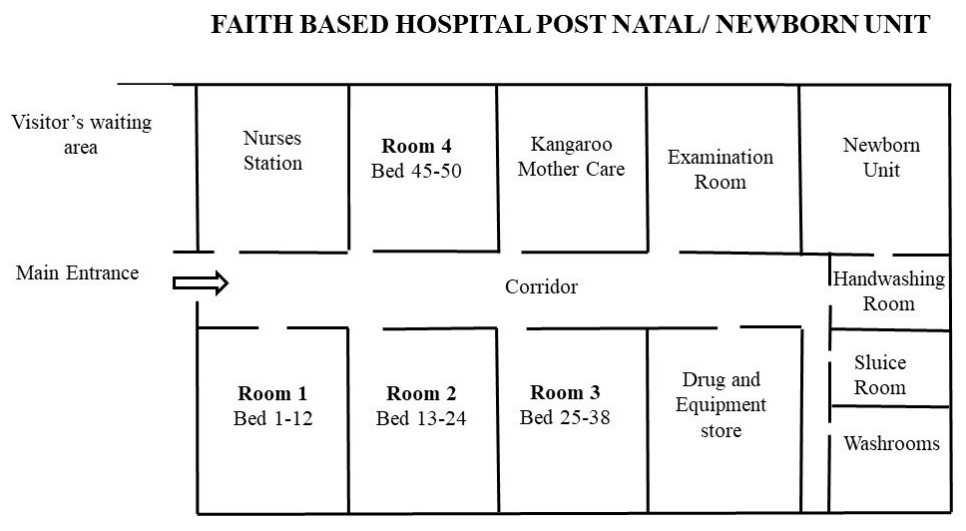
The newborn unit in the faith-based hospital.

The whole post-natal ward is divided into different rooms separated by a wide corridor, as shown in Figure 4.

One side contains five rooms. The first is the nurse station where a ward clerk, cleaners, nurses and, at times, students and clinical officers sit. There are four additional rooms that, in order of occupancy, contain post-natal mothers, kangaroo mother care (KMC) ^1^, the examination room and the NBU. The opposite side of the ward contains three rooms housing post-natal mothers, a store and, at the extreme right end, washrooms and a sluice room. The KMC room has five beds, which were purchased and equipped by the same international non-governmental organisation that equipped the KMC and kangaroo father care rooms at the public hospital. The post-natal ward houses both mothers with well babies and those whose babies have been admitted to the NBU.

***Entry into the NBU.*** Before entry into the NBU, there is a changing room where health workers and mothers change into disinfected flip flop shoes and hang extra clothing. There is also a sink with instructions for washing hands and linen baskets for depositing any dirty linen. The NBU, which is slightly more spacious than the private NHDU, is one room with a multiple patient space of 360ft ^2^ with seven infant incubators. The room has a small desk with two chairs and a desk phone. There are several plastic and metallic chairs for mothers to sit on while breast or cup feeding. There are two sinks with running water, several buckets containing disinfected water, and cups and bowls in one corner of the room. 

The walls are covered with photocopies of standards and regulations of various newborn care procedures. The NBU appears and feels well organised and is always clean, but can get extremely hot and stuffy during feeding times. However, when mothers exit after feeding times, there is always calm and the air is filled with the sound of music from a small radio broadcasting a local religious station.

### Equipment inside the wards

All the three hospitals have basic newborn equipment. The private hospital does not have a designated KMC room; instead, mothers sit on chairs next to the incubators to practice KMC. Table 3 summarises basic infrastructural services in the three newborn units as observed and as told to the first author by the nurse in charge at each NBU.

**Table 3.** Basic equipment and services in newborn units (NBUs).

Description		Hospitals		
		Public	Private	Faith based
Capacity	No. of cots	21	10	0 *
	No. of incubators	11	25	10 (seven in NBU and three in store used during high admissions)
Power	Frequency of power outage	Monthly	Less than monthly	Less than monthly
	Generator to serve NBU	No	Yes	Yes
Basic infrastructure	Heating in NBU	Yes	Yes	Yes
	Running water	Yes 7/10 times needed	Yes	Yes
	Ambulances available	8/10 times needed	Yes, always	Yes, always
Use of incubators	Incubators shared	Yes	No	Yes
Organisation	Separate sick and healthy newborns	Yes	Yes	No
	Most seriously ill newborns nearest the nursing station	Yes	No	No
	Has isolation room	Yes	Yes	Doesn’t admit babies from outside hospital
Refrigeration	Fridges available for mothers to store breastmilk	No	Yes	No

*The nurse in charge mentioned that they encourage newborn contact with mothers for those babies requiring breastfeeding support only.

## Section 2: What nurses perceived as important for providing quality inpatient neonatal care

### A spacious, properly laid out and a clean working space

All nurses mentioned the important role a spacious ward plays in facilitating their delivery of quality care. Although the descriptions above suggest the public and even the private NBUs were overcrowded, it is only nurses in the faith-based hospital who raised issues with the amount of space in the ward.

“First *, this place is very small… We have many incubators in the store but we cannot fit all of them here. This limits the quality of care we can offer here…*” - Faith-based 01

Eight nurses across the hospitals described the importance of a well laid-out ward. For those in the public hospital, a proper layout would go beyond the NBU itself, including a hostel for mothers to sleep in, and a glass-walled area to enable mothers to see their babies in the incubators without necessarily being physically present in the patient area.

*“Mothers should have a hostel next to the NBU, where they can see their babies straight, but currently being housed downstairs makes them anxious because they cannot see their babies without coming upstairs in the ward...” -* Public 03

The nurse in charge in the public hospital NBU suggested that a properly laid-out ward would have rooms located at the front of the ward to enable nurses, mothers and visitors to change into hospital-provided sanitised gowns and shoes before entering the ward.

Nurses in the faith-based hospital emphasized the importance of having the NBU located closer to the birthing area to ease their movement. This was because their NBU was far from theatre and maternity ward.

*“Normally we need a nursery close to a theatre and close to a maternity ward because let’s say we have a baby who has asphyxia and needs to get nursery care, we are disadvantaged in a way so we have to run as you ambu-bag at the same time”* - Faith-based 07

Cleanliness was described by all nurses as important for the promotion of health and wellbeing among nurses as well as for their patients.

“Quality care is when the environment is clean… *spacious*, as a nurse you enjoy working in such a fresh, clean ward….” - Public 03

In the faith-based hospital, a clean environment was described as one that was free from infection and therefore as important as medication in promoting healing and recovery. Observations and interviews showed that nurses cleaned the NBU for this reason, a role that was carried out by cleaners in the other wards in the same hospital.

*“...cleanness NBU is top priority, it is not about the drug, it is about making sure this babies don’t catch infections, so we make sure we clean our floors, incubators, utensils for feeding, and cleaning of linen and everything we make sure NBU is clean for offering quality care …”* - Faith-based 04

In the public hospital, observations showed a sharp contrast between what nurses felt was right and what was observed in terms of cleanliness, which was a challenge. This was because there was one cleaner, who was overwhelmed by their workload, which involved cleaning all floor and wall surfaces, incubators and feeding cups. The cleaner was also responsible for preparing hot water for beverages in the KMC rooms, changing linen and occasionally purchasing personal items for the mothers from shops outside the hospital. Secondly, the lack of regular water supply on the ward contributed to poor cleaning and a foul smell on the ward at times. Although the NBU in the public hospital had been constructed four years ago, observations showed leaking pipes, taps tied with polythene bags, broken toilet cisterns, also affirmed by nurses through interviews.

*“…the ward is smelly and flooded: plumbing was done poorly and most times there is no water, so toilets like today are not flushed, the mothers have not showered, the utensils for the KMC mothers are not washed …” -* Public 09

### Service infrastructure

In all three hospitals there was unanimity among nurses that the delivery of quality care was reliant on the availability of basic medical equipment such as incubators, cots and monitors. During the period of study, observations in the private hospital showed that the ward had enough incubators and monitors and there was no sharing of these facilities by newborns. This was different in both the public and faith-based hospitals, where shortages of incubators were observed, with more than two babies in one incubator.

In addition to the equipment, nurses mentioned the importance of medicines and other materials such as oxygen, linen, fluids, mosquito nets and hand wash to provide quality inpatient care.

*“…materials such as linen, mosquito nets, fluids, oxygen and drugs are important…” - Public* 02

In the public hospital, constant shortages of fluids and oxygen were common; this was observed as well as reported by all the ten nurses. There was a problem of mosquitoes on the ward also. In the private and faith-based hospitals, observations showed that all the necessary consumables were provided for nurses and further replenishment was possible through requisition to the stores with prompt deliveries.

### Staffing and standardisation of practice

Ten nurses mentioned that for them to provide quality care, there was a need for hospitals to hire enough nurses because they experienced fatigue and burnout under the current staffing levels.

*“Quality care is when there are enough nurses to avoid the issue of exhaustion and burnout. When you are continually exhausted from the workload, you cannot deliver quality, work becomes a burden…”* - Faith-based 08

Variations were observed, as well as captured in interviews, in the ways in which the available nurses in each hospital were organised. The public and faith-based hospitals allocated nurses per shift irrespective of workload, while the private hospital allocated nurses according to number and acuity of patients. In the public and faith-based hospitals, two nurses were supposed to be present during the day and one nurse at night. In both the public and faith-based hospitals, nurses expressed their dissatisfaction that these systems were not responsive to either their needs or those of the babies.

*“When you report and find many babies and you are all alone, it is usually tricky and you have to do your best; it is not easy to get relief from other units because they also have their own shortages. You will be required to plan well, prioritize and handle all the cases …”* - Public 01

Even in the much better staffed private hospital where nurses expected to have a ratio of 1 nurse to 2 babies, occasional increases in admissions created understaffing challenges. Unlike the public and faith-based hospitals where there was no additional support, it was the private hospital policy to call locum nurses to cover nursing gaps. However, several of the nurses were concerned with the lack of time available for the appropriate induction of locums and their occasional non-availability.

“… *another challenge is the use of locums…although we have pool nurses, sometimes none of the pool nurses is available when we need them …sometimes we get new ones who are not familiar with the environment had have not been inducted, they can bring quality down* …” - Private 09

Nurses in the public and private hospitals applauded the availability of standards or guidelines of practice, which were displayed on walls for the public hospital and online for the private hospital, to guide the new nurses on how to calculate feeds, fluids and medication. Although not mentioned by nurses in the faith-based hospital, the NBU walls were covered with copies of such guidelines and two nurses who had recently joined the ward made constant reference to them.

### Organisation and delivery of care

Nurses across the sectors mentioned the importance of teamwork and emergency preparedness. They all described team work as exemplified through joint ward rounds and regular consultation between doctors and nurses and between nurses. However, observations in the public hospital showed a lack of participation by nurses in ward rounds, which nurses attributed to an absolute shortage in staffing, making it impossible for them to accompany other healthcare professionals working on the wards.

*“Yes, we have ward rounds, but because of the shortage of nurses, sometimes you are not able to attend…”* - Public 01

In the private and faith-based hospitals, it was observed that nurses and doctors conducted joint ward rounds.

Nurses in private and faith-based hospitals expressed the importance of an availability of senior nurses and doctors for mentorship of and consultation by junior nurses in enhancing teamwork.

*“I think quality care is when a nurse recognizes any problem and the doctor won’t decline to come and even the colleagues if consulted they respond and together they are able to intervene early enough*…” - Private 01

Emergency preparedness in this paper refers to the capacity of a hospital to respond to medical emergencies and although all nurses expressed its importance in enhancing care, there was no internal emergency communication system in the public and faith-based hospitals. It is only the private hospital that had an internal medical response system referred to as code blue.

*“…if there is an emergency, you will hear the emergency call bell and it is possible to know in which department the emergency has occurred…there is a nurse allocated for emergency rescue, we also have a crash cart basically for emergency purpose and every person in the hospital knows how to use it including the doctors…”* Private 09

## Discussion

The aims of this study were to describe the context of newborn nursing care in selected Kenyan NBUs and to document what nurses perceive as requirements for them to provide quality inpatient care. It was an attempt to go beyond the global exercises such as Service Provision Assessments and Service Availability and Readiness Assessments ^19– 21^ that generate health service delivery data by checking indicators of the presence or absence of different forms of infrastructure or resources. Instead, we explore the detailed context of the work environment as experienced by nurses. First, this study has shown that nurses across the sectors have a common thinking of what is required for them to provide quality care to inpatient newborns. Nurses in both public and faith-based hospitals were concerned about a properly laid-out ward, and related aspects such as availability of a hostel for mothers and proximity to the birthing (delivery) area. We observed small overcrowded rooms in the public and faith-based NBUs and the NHDU in the private hospital. These ward areas had between four (private NHDU) and 15 (public hospital) more incubators in the space available in comparison to the minimum recommended standards for newborn ICU design by the American Academy of Paediatrics ^22^. It is only the NICU in the private hospital that met the minimum recommendations for bed space.

We also observed absolute understaffing in both public and faith-based hospitals, with occasional understaffing in the private hospital despite the option to call in locums whenever patient ratios increased. All the nurses were concerned about staffing; the public and faith-based hospital nurses were more concerned about absolute numbers, while those in the private hospital were concerned about the availability and capability of locums. In the public and faith-based hospitals, nurses’ work was further undermined by being on overcrowded and very hot wards, characterised by a lack of equipment and materials sufficient for the workload. These physical elements interact and impact on organisational issues such as teamwork and emergency preparedness. Nurses in the private hospital, despite challenges with insufficient space on the NHDU, worked in a resource rich environment. This provided nurses and physicians opportunities to work as a team, both routinely as part of ward rounds and through well-functioning emergency response systems. This interaction, linked to planning and discussing care together through mechanisms such as multi-professional ward rounds, also helped promote respect for the role of each professional.

For all the nurses, foremost amongst their concerns was access to adequate basic resources including equipment, oxygen, and drugs in order to provide quality care. In general, nurses in the private hospital seemed satisfied with their work environment, unhindered by lack of resources and able to focus on caring for the sick newborns, painting an overall picture of quality care. On the other hand, nurses in the public and faith-based sectors were observed having to spend time developing ‘work-arounds’ because of material and human resource shortages. Whereas the private hospital had more sophisticated resources (ventilators), which was considered a marker of advanced care, the absence of this technology was not considered a threat to quality of care by nurses in public and faith-based hospitals. This is perhaps because these nurses are more concerned about basic resource availability than sophisticated resource availability. Nurses across sectors also indicated that efforts to standardize care in the form of agreed guidelines on medical and nursing processes across sectors could also provide opportunities for broadly based improvement approaches.

In the literature, hospital structures, including both their fixed and moveable components, have been described to have a significant impact on human performance, especially on the health and safety of employees, patients, and families ^23^. Poorly designed wards, such as described in the faith-based hospital where the birthing area is far from the NBU and the lack of accommodation for mothers in the public hospital, show a lack of consideration in their design and construction. This is in spite of the public hospital’s recent construction. To improve the physical built environment, Henriksen and colleagues propose that hospitals need to take a proactive approach to build quality into the design process ^24^. 

Nursing shortages have also been shown to have an effect on quality of care as well as on the workers themselves ^25^. Health workers working in critical care units in the Kilimanjaro area of Tanzania ^26, 27^ reported that their performance was hindered by a shortage of proper equipment and irregular drug supplies. A study that explored barriers in the Delivery of Emergency Obstetric and Neonatal Care in Burundi and Northern Uganda resulted in similar findings ^28^. Another study in over 30 countries showed significant associations between patient-to-nurse ratios and mortality, and that understaffing in critical care wards leads to increased infections and poor outcomes ^29, 30^. Similarly, as has been shown in this study, resource scarcity does affect both nurses and patients, for example, the nurses coped with understaffing by resorting to prioritizing care. A study in Kenyan newborn wards, which aimed to quantify nursing care tasks delivered to sick newborns and identify tasks left undone, reported associations between staffing and nursing care left undone ^31^.

Strikingly absent in this study was a call from nurses for neonatal care training despite having only one nurse with a specialised neonatal nursing qualification. This is in contrast to findings of a multi country analysis that established that inadequate care in facilities is caused by a number of constraints, compounded by a lack of specific knowledge and competencies in dealing with small and sick newborns amongst existing nursing staff ^32^. This could be attributed to the fact that the nurses in the public hospital and faith-based hospitals reported that such specialisation to be expensive and undervalued in their work environments. Observations showed that nurses in the private hospital already had access to similar short trainings through the opportunities in the teaching arm of the university hospital.

Although teamwork in this study was not explored in depth, nurses in the public hospital perceived insufficient nurse numbers as a structural inadequacy as it undermined teamwork. Teamwork, where employees should work together to achieve a common goal, is considered central to any system, including health care ^33^. How health workers organise care through consultation among physicians, nurses and other members of the health care personnel has been shown to not only increase the harnessing of knowledge and skills, but also to contribute to perceived and actual improvement in the quality of care through collective decision making ^34^. However, a study done in a NICU in the Netherlands to understand provider perspectives on what it means to work together showed that apart from different meanings given to teamwork, workplace factors such as staffing and provider characteristics affected working together. Participants in the Netherlands study, just as in this study, noted that hospital rounds in the NICU did not consistently include all team members even though this inconsistency hampered communication and work coordination ^35^.

To strengthen health systems, WHO proposes ‘any array of initiatives and strategies that improves one or more of the functions of the health system and that leads to better health through, among others, quality’ ^36^. It is believed that these initiatives would then have a ripple effect on the health system. The implication is that nursing shortages have to be addressed in order to have influence on processes of care such as ward rounds and emergency preparedness. Whereas this study has shown that nurses have a common understanding of what they need for them to provide quality care to inpatient newborns, in many settings, structural factors lie largely beyond the control of these front-line providers ^37^. Policy makers and planners need to engage much more with such providers to address infrastructure inadequacies and consider during the design process how people work and how families engage in care. In assessing quality, we also need to move away from audit-like checklists of resource that are a very crude and arguably inadequate way of assessing structural elements of quality to paying attention to what nurses require and how they work ^38^.

## Study limitation

A major limitation is that the context in which this study was conducted in addition to perceptions are not static. Therefore, caution needs to be exercised when interpreting these findings. The plurality of the health sector in Kenya with a vast private sector, limits any attempt to generalise the findings as care is needed when refereeing to similar settings.

## Conclusions

Despite the diversity of their work environments, leading to problems that are very different in magnitude and nature, nurses working in newborn wards in Nairobi hold similar ideas of required hospital capacities for them to deliver quality care. Evidence of how nurses operate in these contexts and the support available is critical for providing guidance on how to strengthen their performance in the long term. Nurses as frontline care givers and intervention intermediaries, irrespective of their work contexts, have similar aspirations, needs and expectations from health systems for how they should be supported to provide quality inpatient care for newborns. There is therefore a need to direct efforts to improve quality towards experienced based co-design where we listen to the problems that nurses experience. These problems are about structure, including and especially human resources for health and the consequences of inadequate structure.

## Data availability

### Underlying data

The authors confirm that, for ethical and security reasons, access restrictions apply to the data underlying the findings. The authors are unable to make the data publicly available because of the terms of data sharing included in the consent forms used for this study. Furthermore, neither the hospitals nor the participants can be effectively de-identified in the interview transcripts. However, access will be granted on case-by-case basis upon requests from researchers through the Data Governance Committee of the KEMRI/Wellcome Trust. Such research requests can be sent to the coordinator of the Data Governance Committee at Data_Governance_Committee@kemri-wellcome.org. Access to the underlying data will be granted upon request from a researcher for the purposes of further research once there is a protocol that has been approved by an ethics committee.

### Extended data

Harvard Dataverse: Extended data for: “But you have to start somewhere….”: Nurses’ perceptions of what is required to provide quality neonatal care in selected hospitals, Kenya. https://doi.org/10.7910/DVN/FV5O1I ^16^

This project contains the following underlying data:

- Interview guide.docx
- Wellcome open COREQ_checklist.pdf

Data are available under the terms of the Creative Commons Zero “No rights reserved” data waiver (CC0 1.0 Public domain dedication).

## Note


^1^The WHO has defined KMC as early, continuous, and prolonged skin–to–skin contact (SSC) between the mother and preterm babies; exclusive breastfeeding or breast milk feeding; early discharge after hospital–initiated KMC with continuation at home; and adequate support and follow–up for mothers at home Chan, G. J., Valsangkar, B., Kajeepeta, S., Boundy, E. O., & Wall, S. (2016).
