# Supplementary material for: “But you have to start somewhere….”: Nurses’ perceptions of what is required to provide quality neonatal care in selected hospitals, Kenya
Source: Wellcome Open Res. 2020 Feb 17;4:195. Originally published 2019 Dec 6. [Version 2] doi: 10.12688/wellcomeopenres.15592.2 (PMC7309418; doi:10.12688/wellcomeopenres.15592.2)
Supplement: Peer review history

# Peer review: Reviewer response for version 2

**DOI:** 10.21956/wellcomeopenres.17261.r37934

**Published:** 2020-06-18

**Authors:** O'Brien Munyao Kyololo

**Affiliations:** 1 School of Nursing, Moi University, Eldoret, Kenya

I have read through the revised version of the manuscript and I would like to make the following observations:

**Sentence structure**

- Abstract: The last sentence of the “Results” section is ambiguous; should be revised.
- The last sentence under “Study settings” requires revision to bring out the message clearly.

**Data collection**

 It is indicated that 29 interviews were conducted but only 26 were audio-recorded. The authors should indicate why the 3 interviews were not audio-recorded and whether they were part of the analysis.

**Data analysis **

- The authors have not provided a detailed account of how the coding structure/matrix was developed.
- Considering that the authors only used NVIVO for analysis, then the need to suggest other softwares (i.e., FreeQDA) to the readership does not arise.

**Results**

 Although it is reported that thematic analysis was employed in data analysis, neither themes nor sub-themes are reported. Furthermore, reading through the findings suggests that the two levels of thematic analysis (latent and manifest) were not carried out. The authors appears to have predominantly conducted manifest analysis. That being the case, it would mean that the authors employed content analysis approach.

Is the work clearly and accurately presented and does it cite the current literature?

Yes

If applicable, is the statistical analysis and its interpretation appropriate?

Not applicable

Are all the source data underlying the results available to ensure full reproducibility?

Yes

Is the study design appropriate and is the work technically sound?

Yes

Are the conclusions drawn adequately supported by the results?

Yes

Are sufficient details of methods and analysis provided to allow replication by others?

Partly

Reviewer Expertise:

Neonatal care in Low and middle income countries.

I confirm that I have read this submission and believe that I have an appropriate level of expertise to confirm that it is of an acceptable scientific standard.

# Peer review: Reviewer response for version 1

**DOI:** 10.21956/wellcomeopenres.17075.r37675

**Published:** 2020-02-11

**Authors:** Carole Kenner

**Affiliations:** 1 School of Nursing, Health, and Exercise Science, The College of New Jersey, Ewing, NJ, USA

This is a very important topic. It aligns with the SDGs, especially SDG #3 *“Ensure healthy lives and promote well-being for all at all ages”. *The rationale for selecting Kenya as a site due to its high neonatal mortality rate is explained. Literature cited is appropriate and the references are up to date. Workforce and quality themes are supported by the data and align with studies in other countries. The data collection was explained, however if a structured interview was used then how many questions were asked? Any recommended changes in the questions now that the study is complete?

 The statement of what the new findings imply could be expanded beyond listening to nurses and to delve more deeply into the issues impacting quality care and neonatal outcomes. This still can be from the nurses’ perspectives but requires more direct questions about equipment, medications, staffing-including sleep deprivation and burnout, skills, training, cleanliness, space constraints, and interprofessional teamwork.

 It would be helpful to have a statement indicating how representative your selected hospitals were of others found in Kenya.

 There is enough information gained from the study that it could be replicated in other countries. The write up might be strengthened if at the end there was a recommendation to also look at training/education of staff in future studies. This factor also impacts on quality care and neonatal morbidity and mortality.

Is the work clearly and accurately presented and does it cite the current literature?

Yes

If applicable, is the statistical analysis and its interpretation appropriate?

Not applicable

Are all the source data underlying the results available to ensure full reproducibility?

Yes

Is the study design appropriate and is the work technically sound?

Yes

Are the conclusions drawn adequately supported by the results?

Yes

Are sufficient details of methods and analysis provided to allow replication by others?

Yes

Reviewer Expertise:

Neonatal palliative care, health policy, workforce, transition from hospital to home; perinatal substance abuse.

I confirm that I have read this submission and believe that I have an appropriate level of expertise to confirm that it is of an acceptable scientific standard.

## References

[1] : Impact of Training on Nurses Performance and Productivity at Neonatal Intensive Care Unit (NICU). *JOJ Nursing & Health Care*.2018;9(2) : 10.19080/JOJNHC.2018.09.555758 10.19080/JOJNHC.2018.09.555758

# Response

**Published:** 2020-02-13

**Authors:** Mary NYIKURI

**Affiliations:** KEMRI-Wellcome, Kenya

Thank you for taking your time to review and provide such useful feedback for strengthening this paper.

REVIEWER 2

**Reviewer comment 1**

This is a very important topic. It aligns with the SDGs, especially SDG #3 *“Ensure healthy lives and promote well-being for all at all ages”. *The rationale for selecting Kenya as a site due to its high neonatal mortality rate is explained. Literature cited is appropriate and the references are up to date. Workforce and quality themes are supported by the data and align with studies in other countries.

**Author response**. Thank you so much.

**Reviewer comment 2**:  The data collection was explained, however if a structured interview was used then how many questions were asked?

**Author Response**: The interview guide had 15 main questions.

**Reviewer comment 3**: Any recommended changes in the questions now that the study is complete?

**Author Response:** We note that after the study ended, we would recommend additional questions on what nurses perceive as necessary interventions to help them deliver quality care to inpatient newborns.

**Reviewer comment 4**: The statement of what the new findings imply could be expanded beyond listening to nurses and to delve more deeply into the issues impacting quality care and neonatal outcomes. This still can be from the nurses’ perspectives but requires more direct questions about equipment, medications, staffing-including sleep deprivation and burnout, skills, training, cleanliness, space constraints, and inter professional teamwork.

**Author Response**: We agree with your suggestion and the sentence has been expanded to read as follows: Interventions and investments to improve quality need to be directed towards experienced based co-design where we listen to the things that nurses tell us are problems such as infrastructure, staffing, and interprofessional teamwork if we are to improve hospital services for newborns. 

**Reviewer comment 5: **It would be helpful to have a statement indicating how representative your selected hospitals were of others found in Kenya.

**Author Response** : We have added the following information on the section of study setting. “With devolution of health services, where the county governments are responsible for service delivery, the hospitals are as diverse as the leadership and governance systems in the counties. However, most public hospitals are characterised by overcrowding and high patient to nurse ratios. The Private hospital represents majority of high end private hospitals where facilities are well maintained, no overcrowding and sufficient staffing levels. The faith based hospital on the other hand is not representative of faith based hospitals as this sector is as diverse as the founder and funders.

**Reviewer comment 6**: There is enough information gained from the study that it could be replicated in other countries. The write up might be strengthened if at the end there was a recommendation to also look at training/education of staff in future studies. This factor also impacts on quality care and neonatal morbidity and mortality.

**Author Response**: Thank you for this suggestion. We have published the findings on training and mentorship elsewhere below:

**Nyikuri M**, Kumar P, English M, Jones C. “I train and mentor, they take them”: A qualitative study of nurses' perspectives of neonatal nursing expertise and its development in Kenyan hospitals. Nursing Open. 2020;00:1–9. https ://doi.org/10.1002/nop2.442

# Peer review: Reviewer response for version 1

**DOI:** 10.21956/wellcomeopenres.17075.r37309

**Published:** 2020-01-07

**Authors:** O'Brien Munyao Kyololo

**Affiliations:** 1 School of Nursing, Moi University, Eldoret, Kenya

Thank you for the opportunity to review manuscript titled: “But you have to start somewhere…”nurses’ perceptions of what is required to provide quality neonatal care in selected hospitals, Kenya”. The study sought to explore nurses’ understanding of quality of newborn care by conducting one-on-one interviews triangulated with observations and informal dialogue with key informants. The study is important considering the limited literature on the quality of newborn care, from the perspective of front-line staff, in sub-Saharan Africa where most of the global neonatal morbidity and mortality occur.

 Generally, the paper is well written; the research question is well defined, the methods are clearly described and the key findings are highlighted. There are, however, areas and/or suggestions that need to be addressed in order to improve the scholarly appeal of the paper.

**Sentence structures:**

 In a number of instances, the authors have used ambiguous and often long sentences thus making it difficult for the reader to follow through the arguments. For instance, the last sentence under “Ethical statement” could be revised to read *“All participants provided voluntary written consent before being interviewed”. *Similarly, the 2 ^nd^ sentence of paragraph two in the “Results” section, the authors have used a 6-line sentence which is very challenging for the reader to read through.

 Additionally, there is a lot of repetition in the findings section particularly when making reference to figures. Perhaps the authors could consistently make reference to the figures in parenthesis (e.g., Figure 1) instead of putting them as part of the sentence structures throughout the manuscript.

 There are also numerous typographical errors throughout the manuscript which should be corrected.

**Study setting**

 It would be important to indicate the level of newborn care of each of the study sites according to universally accepted classification (e.g. according to the American Academy of Pediatrics).

**Data collection**

 It is important that the authors provide a detailed description of the sample rather than describing the sample size in this section. Since this was a qualitative study, it is expected that the researchers will only know about the sample size upon attaining data saturation.

 Who did the transcription of the data? And what level of training/expertise did the transcriptionist have?

 Was there any changes to the interview guide following the pilot study? And if not, was the pilot data used as part of the main study data?

 It is also important for the researchers to describe how the Swahili Transcripts were translated into English and what measures were put in place to maintain quality of data during translation.

**Data analysis**

 The process of developing the coding structure should be clearly described.

 The authors report thus *“Other open source software such as FreeQDA could also be used”.* Was the software used or not? It is important for this to be stated for clarity.

 It is important to include a sub-heading/content on ***“Rigour”*** after the data analysis.

**Results**

 The demographic characteristics of the interviewees should be the first part of the section.

 Making reference to the recommended spacing norms in newborn units is not part of the findings and, thus, should be moved to the appropriate section of the manuscript - Discussion section *(e.g., this is in comparison to the minimum recommended floor space of 120 ft ^2 ^per infant with 8ft spacing between bed ^18^).*

 Although it is reported that thematic content analysis was used in data analysis, the themes and sub-themes arising from the analysis are not reported. Rather, it appears that the researchers employed simple content count in data analysis.

 Furthermore, the data is reported in reference to the study hospitals (public, private, faith-based). To show congruence (and divergence when present) of views, all data ought to have been analysed as one unit and reported as such. To demonstrate that there were data from the different units, the researchers should provide a little bit more details about the sources of their verbatim quotes in the findings *(e.g. a diploma nurse in a private hospital).*

 On two occasions ***(Staffing and standardisation of practice AND Organisation and delivery of care),*** the authors have provided 2 verbatim quotes for a results category. Only one verbatim quote should be used for each “area” of reporting.

**Discussion**

 In the first 3 paragraphs of the discussion, the authors have mainly provided their interpretation of their findings rather than striving to situate the study results in the context of the existing literature.

**Additional content required**

 The authors should also include sections on:

1. Implications for Policy, Practice and Research.
2. Study limitation.

Is the work clearly and accurately presented and does it cite the current literature?

Yes

If applicable, is the statistical analysis and its interpretation appropriate?

Not applicable

Are all the source data underlying the results available to ensure full reproducibility?

Yes

Is the study design appropriate and is the work technically sound?

Yes

Are the conclusions drawn adequately supported by the results?

Yes

Are sufficient details of methods and analysis provided to allow replication by others?

Partly

Reviewer Expertise:

Neonatal care in Low and middle income countries.

I confirm that I have read this submission and believe that I have an appropriate level of expertise to confirm that it is of an acceptable scientific standard, however I have significant reservations, as outlined above.

# Response

**Published:** 2020-01-22

**Authors:** Mary NYIKURI

**Affiliations:** KEMRI-Wellcome, Kenya

Thank you so much for taking your time to review this article. I am taken note of the comments which will go a long way in strengthening the paper. I am revising the article as I incorporate your feedback where necessary. 

Kind regards

Mary

# Response

**Published:** 2020-02-13

**Authors:** Mary NYIKURI

**Affiliations:** KEMRI-Wellcome, Kenya

REVIEWER 1

Thank you for the opportunity to review manuscript titled: “But you have to start somewhere…” nurses’ perceptions of what is required to provide quality neonatal care in selected hospitals, Kenya”. The study sought to explore nurses’ understanding of quality of newborn care by conducting one-on-one interviews triangulated with observations and informal dialogue with key informants. The study is important considering the limited literature on the quality of newborn care, from the perspective of front-line staff, in sub-Saharan Africa where most of the global neonatal morbidity and mortality occur.

Generally, the paper is well written; the research question is well defined, the methods are clearly described and the key findings are highlighted. There are, however, areas and/or suggestions that need to be addressed in order to improve the scholarly appeal of the paper.

**Reviewer comment 1: Sentence structures:**

In a number of instances, the authors have used ambiguous and often long sentences thus making it difficult for the reader to follow through the arguments. For instance, the last sentence under “Ethical statement” could be revised to read *“All participants provided voluntary written consent before being interviewed”. *Similarly, the 2 ^nd^ sentence of paragraph two in the “Results” section, the authors have used a 6-line sentence which is very challenging for the reader to read through.

**Author Response** **:** We have noted your suggestions and have revised the sentence under ethical statement as below:

All participants provided voluntary written consent before being interviewed. The second sentence of paragraph two in the results section has now been broken down into two sentences as below; We first present in section I, the observed work environment, the real picture of the spaces where nurses work and the variation across these contexts. A variation that can easily be missed through inventories that may classify very different places as similar on the basis of the list of ‘items’ they contain

**Reviewer comment 2:** Additionally, there is a lot of repetition in the findings section particularly when making reference to figures. Perhaps the authors could consistently make reference to the figures in parenthesis (e.g., Figure 1) instead of putting them as part of the sentence structures throughout the manuscript.

**Author Response**: The authors have taken your suggestion into account and all the figure references have been put in parenthesis.

**Reviewer comment 3**: There are also numerous typographical errors throughout the manuscript which should be corrected.

**Author response** : Thank you, we have noted and corrected areas where this occurred.

**Reviewer comment 4: Study setting**

It would be important to indicate the level of newborn care of each of the study sites according to universally accepted classification (e.g. according to the American Academy of Pediatrics).

**Author respons** **e**: We have taken note of this and added a sentence before data collection to describe the levels of care.

**Reviewer comment 5: Data collection**

It is important that the authors provide a detailed description of the sample rather than describing the sample size in this section. Since this was a qualitative study, it is expected that the researchers will only know about the sample size upon attaining data saturation.

**Author response**: The authors have taken note of this. However, this is a report of what already happened [the study has already been completed], the authors describe the sample after saturation was reached.

**Reviewer comment 6:** Who did the transcription of the data? And what level of training/expertise did the transcriptionist have?

**Author response**: We have included the transcriber who was the first author. She is an anthropologist with over 10 years’ experience in transcribing

**Reviewer comment 7:** Was there any changes to the interview guide following the pilot study? And if not, was the pilot data used as part of the main study data?

**Author response**: Thank you for this feedback. There was no pilot study preceding the main study. Instead, MN spent a few weeks in the facilities to try and understand the layout and as well as to refine the interview guide.

**Reviewer comment 8**: It is also important for the researchers to describe how the Swahili Transcripts were translated into English and what measures were put in place to maintain quality of data during translation.

**Author response** : Thank you for this comment. We have included information on how this was done. It reads in the main paper as indicated below:

“To maintain quality of data during translation, MN did careful comparison with original questions and against other transcripts. In addition, MN carried out back translation to ensure that original meaning was maintained”

**Reviewer comment 9: Data analysis**

The process of developing the coding structure should be clearly described.

**Author Response** : We have clarified that section by rephrasing to read as below:

The first author MN, carried out analysis by open coding using Nvivo 10 software ^15^. Initial codes were generated from the interview guide whilst emerging themes were subsequently analysed in relation to relevant literature culminating into thematic analysis

**Reviewer comment 10**: The authors report thus *“Other open source software such as FreeQDA could also be used”.* Was the software used or not? It is important for this to be stated for clarity.

**Author response** **:** Thank you for the suggestion. Free QDA was not used, instead NVIVO 10 was used but we provide that alternative in case a reader or potential author is interested in re-analysing the data and have no access to NVIVO which is not free, then they can use FreeQDA.

**Reviewer comment 11**: It is important to include a sub-heading/content on ***“Rigour”*** after the data analysis.

**Author response**: We have added this section as per your suggestion.

**Rigour: **To ensure data quality, and analytic rigour, the interviews were first triangulated with observation data; secondly by feedback to the nurses for sense checking; thirdly, the researcher made presentations in various local forums where feedback was received and incorporated

**Reviewer comment 12: Results**

The demographic characteristics of the interviewees should be the first part of the section.

**Author response**: We have revised this section to put demographic characteristics as the first part of the results section.

**Reviewer comment 13:** Making reference to the recommended spacing norms in newborn units is not part of the findings and, thus, should be moved to the appropriate section of the manuscript - Discussion section *(e.g., this is in comparison to the minimum recommended floor space of 120 ft* *^2^* *per infant with 8ft spacing between bed* *^18^* *).*

**Author response**: The authors have rectified this as per your suggestion.

**Reviewer comment 14**: Although it is reported that thematic content analysis was used in data analysis, the themes and sub-themes arising from the analysis are not reported. Rather, it appears that the researchers employed simple content count in data analysis. Furthermore, the data is reported in reference to the study hospitals (public, private, faith-based). To show congruence (and divergence when present) of views, all data ought to have been analysed as one unit and reported as such. To demonstrate that there were data from the different units, the researchers should provide a little bit more details about the sources of their verbatim quotes in the findings *(e.g. a diploma nurse in a private hospital).*

**Author respons** e: Thank you for this suggestion. We aimed at looking at perceptions of quality of care by nurses working in different sectors. We therefore analysed the data as one unit but bring out the congruence and divergence using verbatim quotes from the different sectors.

**Reviewer comment 15:** On two occasions ***(Staffing and standardisation of practice AND Organisation and delivery of care),*** the authors have provided 2 verbatim quotes for a results category. Only one verbatim quote should be used for each “area” of reporting.

**Author response:**  Thank you, this has been addressed in the paper by deleting one quote.

**Reviewer comment 16: Discussion**

In the first 3 paragraphs of the discussion, the authors have mainly provided their interpretation of their findings rather than striving to situate the study results in the context of the existing literature.

**Author response**: We agree that this is how the section is presented. This is to deliberately remind the reader of what the main tenets of the paper are before situating the findings in wider literature.

**Reviewer comment 17: Additional content required**

Implications for Policy, Practice and Research. & Study limitation

**Author response:**  We have taken note of your suggestion. We however consider the conclusion section to make reference to implications of this research for different stakeholders.

We have included a study limitation section in the revised paper and it reads  “A major limitation is that context in which this study was conducted in addition to perceptions are not static. Therefore, caution needs to be exercised when interpreting these findings. The plurality of the health sector in Kenya with a vast private sector, limits any attempt to generalize the findings as care is needed when refereeing to similar settings.”
